# Epidermal growth factor receptor gene mutation status in pure squamous-cell lung cancer in Chinese patients

**DOI:** 10.1186/s12885-015-1056-9

**Published:** 2015-03-01

**Authors:** Qing Zhang, Lei Zhu, Jie Zhang

**Affiliations:** Department of Pathology, Shanghai Chest Hospital, Shanghai Jiao Tong University, Shanghai, China

**Keywords:** Lung cancer, Squamous cell lung cancer, Epidermal growth factor receptor gene mutation, *EGFR* gene mutation, Tyrosine kinase inhibitor

## Abstract

**Background:**

Although new individual treatments continue to reshape the landscape of clinical care in patients with lung cancer, most of the progress has been mainly of benefit to patients with lung adenocarcinomas rather than squamous cell lung carcinoma (SQCLC). Our study aimed to determine whether the epidermal growth factor receptor (*EGFR*) gene mutation is present in pure SQCLC. We further determined the mutation frequency and the clinical and pathological features of groups that are in high risk for *EGFR* mutation.

**Methods:**

A total of 185 Chinese patient specimens diagnosed as SQCLC in the Shanghai Chest Hospital at the Shanghai Jiao Tong University were selected for this study. Hematoxylin-eosin stained slides for all cases were reviewed and evaluated by immunohistochemical analysis with the aim of selecting samples with pure SQCLC. After screening, 22 cases were eliminated and 163 pure SQCLC cases remained. Amplification Refractory Mutation System was used to detect the *EGFR* gene mutation status in the 163 SQCLC specimens.

**Results:**

A total of 28 cases with *EGFR* mutation were detected among the 163 specimens. The *EGFR* mutation rate was 17.2% (28/163). Sex and smoking status were significantly associated with the status of *EGFR* gene mutation (*P* = 0.022 and *P* = 0.049, respectively). Age and degree of differentiation were not significantly correlated to *EGFR* mutation (*P* = 0.730 and *P* = 0.651, respectively).

**Conclusions:**

*EGFR* mutations are present in pure SQCLC, which are more frequently detected in females and nonsmoker patients. Our results indicate the importance for all patients with SQCLC to have *EGFR* mutation status examined. These patients with activating *EGFR* mutation could accept tyrosine kinase inhibitors (TKIs) treatment.

**Electronic supplementary material:**

The online version of this article (doi:10.1186/s12885-015-1056-9) contains supplementary material, which is available to authorized users.

## Background

Our understanding of the molecular mechanisms that underlie the development of non-small cell lung carcinoma (NSCLC) has increased significantly during the last decade. Although new discoveries continue to reshape the landscape of clinical care, most of the progress has been mainly of benefit to patients with adenocarcinomas of the lung [1]. The 45th American Society of Clinical Oncology (ASCO2009) stated that epidermal growth factor receptor (*EGFR*) tyrosine kinase inhibitors (TKIs) should be the treatment of choice for advanced NSCLC patients with *EGFR* mutations. A recent study showed that squamous cell lung cancer (SQCLC) accounts for 20–30% of NSCLC [2]. Among the major histological subtypes of NSCLC, the study of the molecular abnormalities in SQCLC has recently started to make minor progress [3]. However, there is still a lack of effective targeted therapy for SQCLC.

The current consensus in the medical community is that *EGFR* mutations are predominantly found in lung adenocarcinoma patients who are Asian, female, and non- or mild smokers [4]. The National Comprehensive Cancer Network (NCCN) 2012 guidelines for NSCLC treatment stated that *EGFR* mutation analysis and ALK gene rearrangement detection should not be routinely recommended for SQCLC [5]. However, in 2011, Tseng et al. conducted a retrospective study, in which 92 patients with advanced SQCLC and unknown *EGFR* mutation status were treated with erlotinib. The results showed an overall response rate (ORR) of 17.4% and a disease control rate (DCR) of 27.2%. Progression-free survival (PFS) and overall survival (OS) were both longer in patients with disease control than in those with progressive disease (7.8 vs. 1.3 months and 20.7 vs. 2.7 months, respectively; *P* < 0.0001 for both). From the results of the study, researchers concluded that a significant proportion of SQCLC patients could benefit from erlotinib treatment, and thus evaluation of *EGFR* mutation status would be necessary [6]. However, the *EGFR* mutation rate in resected SQCLC specimens is 0–7.4% [7,8] and 1–15% in biopsied SQCLC specimens [9]. These rates are much lower than the 42.7% (33.5–56.8%) rate found in lung adenocarcinomas [10]. Adenocarcinoma compositions could alter the *EGFR* mutation status of SQCLC, so whether the relevant mutation in SQCLC samples is caused by the inclusion of adenocarcinoma compositions is controversial. Thus far, few studies have shown *EGFR* mutation in SQCLC, and patients with activating *EGFR* mutation responded to TKIs treatment [11].

Thus, the primary objective of this study was to enhance the diagnostic accuracy for SQCLC using hematoxylin-eosin (H&E) and immunohistochemical (IHC) analyses. The non-SQCLC component was excluded to determine the presence of *EGFR* gene mutation in pure SQCLC. The pathological and clinical features of patients with the *EGFR* gene mutation status are also summarized. The secondary objective of this study was to examine the response of patients, with pure SQCLC and an *EGFR* mutation, to TKIs treatment.

## Methods

### Study samples

Tumor specimens were obtained from 185 Chinese patients with SQCLC that were surgically resected in the Shanghai Chest Hospital at Shanghai Jiao Tong University (Shanghai, China) between June 2006 and June 2012. A total of 119 males and 66 females, with a median age of 62.4 years, were included in the study. The research has been approved by the Ethic Committee, Shanghai Chest Hospital, Shanghai Jiao Tong University, and the approval is added as in Additional file 1. All patients provided written informed consent, and one of patient’s informed consent is added as in Additional file 2.

### Study methods

#### Sample selection

H&E stained specimen slides were read by two experienced pulmonary pathologists and the diagnoses were made according to the 2004 WHO classification system for lung carcinoma. Each pathologist classified the tumor specimens independently and unanimous agreement was obtained. Samples were obtained from four different regions of each tumor. To exclude mixed, non-SQCLC tumor compositions, the highest differentiated sections in the tumors were selected for IHC analysis and DNA extraction. The differentiation of SQCLC was categorized as follows: well differentiated, more than 50% of obvious keratin pearl or intercellular bridge observed in tumor tissues; moderately differentiated, 20–50% of keratin pearl or intercellular bridge observed in tumor tissues; and poorly differentiated, less than 20% of keratin pearl or intercellular bridge.

#### IHC analysis

TTF-1 (Clone 8G7G3/1, DakoCytomation, Glostrup, Denmark), CK7 (Clone OV-TL 12/30, DakoCytomation), ΔNP63 (P40, Calbiochem, California, USA) and high molecular cytokeratin (Clone 34βE12, DakoCytomation) were used for examination of the pathological histology that had been identified through H&E staining to exclude mixed and inconspicuous, adenocarcinoma components. Tissue sections were deparaffinized using xylene and dehydrated in alcohol. Antigen-retrieval was performed by heating the slides in 0.1 M sodium citrate (pH 6.0) for 10 min. Tissue slides were incubated in 0.3% H_2_O_2_ for 10 min to block endogenous peroxidase activity. Sections were incubated with primary antibody for 30 min at room temperature. Sections incubated with antibody diluents (Immunologic, Duiven, Netherlands) were used as the negative control. Sections were developed using the Dako EnVision™ visualization system (DakoCytomation). Pretreatment conditions and antibody dilutions are available upon request.

#### DNA extraction and mutation test

**Figure 1:**
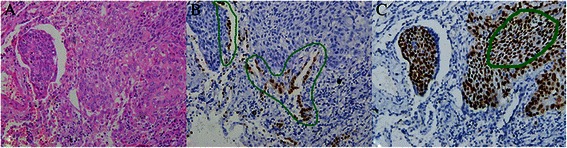
**Method for obtaining pure SQCLC tissues for detection of*EGFR*gene mutation. [A]** HE stained slice diagnosed as SQCLC. **[B]** TTF-1 staining of the residual alveolar epithelium. When obtaining the SQCLC tissues for mutation analysis, the positive regions (indicated by green borders) were avoided. **[C]** ∆NP63 staining. The region outlined by the green border was used to detect the EGFR gene mutation status.

The pure squamous cell cancers were determined by IHC analysis results. ∆NP63 and 34βE12 were positive, and TTF-1 and CK7 were negative. The area of residual, alveolar epithelium was avoided when acquiring the sample tissues (Figure 1) to ensure that the *EGFR* gene mutation was present in pure SQCLC. DNA was extracted from five serial slices of a 5-μm paraffin section using the DNA FFPE Tissue Kit (Qiagen, Hilden, Germany) following the manufacturer’s protocol. The highly sensitive method Amplification Refractory Mutation System (ARMS) (ADx-EG01, Xiamen, China) was used to detect the *EGFR* gene mutation status [12,13].

#### Statistical methods

Univariate analysis was conducted on sex, age, smoking status and the degree of differentiation using Fisher’s exact test to evaluate the effects of clinical and pathological factors relating to the patients and disease characteristics. All statistical tests were done using SPSS17.0 software (http://www.spss.com, IBM Corp., Chicago, IL). Two-tailed t-tests were used and *P* values < 0.05 were considered significant.

## Results

### H&E staining and IHC analysis

**Figure 2:**
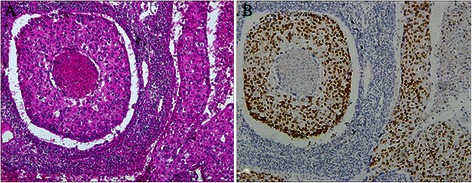
**A poorly differentiated adenocarcinoma with squamous morphology. [A]** The HE stained specimen from this case could be diagnosed as poorly differentiated SQCLC without IHC analysis. **[B]** Positive TTF-1 stained specimen from the same case.

A total of 185 surgical SQCLC samples were examined. Ten samples were excluded after H&E staining owing to the SQCLC sample containing less than a 10% portion of mixed adenocarcinoma, or lymphoepithelioma-like carcinoma, or basal-like large cell carcinoma that had been diagnosed as SQCLC. An additional 12 samples were excluded owing to IHC results. In some cases, all results were negative after analysis using the primary antibodies ΔNP63 (P40), TTF-1, CK7 and 34βE12, and the cases should be diagnosed as large cell carcinoma. In other cases, the results of ΔNP63 (P40) and 34βE12 were negative but positive for TTF-1 and CK7, and the cases should be diagnosed as poorly differentiated adenocarcinoma (Figure 2). The remaining 163 cases were enrolled in the study to evaluate the *EGFR* gene mutation status in pure SQCLC.

A total of 114 (69.9%) males and 49 (30.1%) females with a median age of 61.3 years (range 33–81 years) were enrolled. Cases were divided into three groups based on age, and included 11 cases 30–49 years of age, 123 cases 50–69 years of age and 29 cases ≥70 years of age. In total, 59 cases were considered as well differentiated, 56 moderately differentiated and 48 poorly differentiated. A total of 78 patients were non-smokers, 61 patients were smokers and in 24 cases, smoking status was unknown.

### *EGFR* gene mutation analysis

An *EGFR* gene mutation was detected in 28 of the 163 samples, giving a mutation rate of 17.2% (Table 1). There were 16 cases with 19del mutation and 11 cases with 21L858R mutation, and one 64 years old female smoking patient with poorly differentiation SQCLC harbored both 19del and 21L858R mutations. Sex and smoking status were significantly associated with the rate of *EGFR* gene mutation (*P* = 0.022 and *P* = 0.049, respectively). Age and degree of differentiation were not significantly correlated to *EGFR* gene mutation (*P* = 0.730 and *P* = 0.651, respectively).

**Table 1:** **Baseline demographic, clinical characteristics and the corresponding** ***EGFR*** **gene mutation ratio**

	Positive	Mutation type		Negative	Total	Mutation ratio	*P*value
		19del	21L858R				
		17	12				
**Sex**							0.022
Male	14	12	2	100	114	12.3%	
Female	14	5	10	35	49	28.6%	
**Degree of differentiation**							0.651
Well	10	6	4	49	59	16.7%	
Moderately	8	2	6	48	56	14.3%	
Poorly	10	9	2	38	48	20.8%	
**Age**							0.730
30-49 years	2	2	0	9	11	26.2%	
50-69 years	23	13	11	100	123	11.5%	
≥70 years	3	2	1	26	29	12.5%	
**Smoking status**							0.049
Non-smoking	16	10	6	45	61	26.2%	
Smoking	9	5	5	69	78	11.5%	
Unknown	3	2	1	21	24	12.5%	

## Discussion

*EGFR*-TKIs have been proven to be effective for treating NSCLC cases with *EGFR* mutations. Therefore, many studies have focused on patients with the presence of active *EGFR* mutations, especially those with adenocarcinoma histology. Thus far, studies involving *EGFR* gene mutations and the response to TKIs treatment in SQCLC patients with these mutations have been very few [11] and there has been little consensus in the results. In 2014, Kim et al. reported that an EGFR mutation rate of 6% in SQCLC patients [3]. In 2011, researchers from the Netherlands carried out a multicenter study and found no *EGFR* gene mutation in SQCLC patients [7]. In 2010, Japanese researchers found that the rate of *EGFR* gene mutation in resected SQCLC specimens was 7.4% [8]. In 2007, researchers in Korea found a response to TKI treatment in 69 patients with advanced SQCLC and an *EGFR* mutation rate up to 15% (3/20) [9]. In addition, their results indicated that *EGFR* gene mutation could be the predictive factor for the response to TKIs, although the efficacy was lower for SQCLC than for adenocarcinoma. In the current study, IHC analysis was used to select only pure SQCLC to help elucidate whether the *EGFR* gene is mutated in SQCLC and, if so, to determine the mutation rate, as well as the clinical and pathological features in high risk groups. In 2010, William Travis pointed out that there are some limitations on samples obtained via bronchoscopy or transthoracic needle biopsy when making diagnoses based on pathology and when detecting genomic alterations, owing to the high degree of heterogeneity in lung cancers [14]. Thus, only surgical SQCLC specimens were included in the current study. In addition, primary antibody P40 was used instead of P63, due to its higher specificity when confirming squamous cell cancers [15,16].

In our study, we found a total rate of 17.2% for the *EGFR* gene mutation in patients with SQCLC. This rate was higher than that found in our previous analyses (0–15%) [3,7-9]. There could be several possible explanations for the different results. One possibility could reflect the different sensitivities among the different methods used for analyses. Most of the former studies detected the *EGFR* gene mutation by direct DNA sequencing, which has a sensitivity of only 70%, much lower than the 99% sensitivity of ARMS [12,13]. Thus, the different detection assay could interfere with the mutation relevance rate. A second explanation may be owing to the difference in the sex ratio of the enrolled patients. Because the incidence of SQCLC is lower in females, as also evident in the current study, fewer females were included. The current study showed that the rate of *EGFR* gene mutation in females with SQCLC was 28.6%, which is significantly higher than in males (12.3%) (*P* = 0.022). As a result, we speculate that the total rate of mutation in SQCLC might increase as the number of female cases increases. A third explanation may involve differences among races and regions, as the factors that drive genomic alteration between races are consequential [17,18]. For example, the K-ras gene mutation is higher in Caucasian NSCLC patients than in Asian NSCLC patients. Similarly, Asian patients with adenocarcinoma also have a higher incidence of the *EGFR* gene mutation than Caucasian patients. Furthermore, all of our samples are Chinese patients, which may lead to a different result from other Asian or Caucasian patients.

We found that sex and smoking status were significant factors in the *EGFR* gene mutation rate in SQCLC (female: 28.6% and male: 12.3%, *P* = 0.022; non-smoking: 26.2%, moking: 11.5% and unknown smoking status: 20.8%, *P* = 0.049). However, age and the degree of differentiation had no affect on the mutation rate. To our knowledge, a similar conclusion has not been reported thus far. If further research finds it to be similar to the *EGFR* gene mutation found in adenocarcinoma among high-risk groups, oncologists should consider testing female and non/mild smoking SQCLC patients for *EGFR* gene mutation status and begin TKIs treatment if appropriate.

In 2011, Shukuya et al. [19] carried out a pooled analysis of published reports regarding the efficacy of gefitinib for use in non-adenocarcinoma, non-small cell lung cancer patients harboring *EGFR* mutations. The analysis showed that gefitinib is less effective in non-adenocarcinoma NSCLC harboring *EGFR* mutations than in adenocarcinoma harboring *EGFR* mutations (response rate (RR): 27% vs. 66%, P = 0.000028; DCR: 67–70% vs. 92–93%, *P* = 0.000014; median progression-free survival (mPFS): 3.0 vs. 9.4 months, *P* = 0.0001). We could not obtain adequate data regarding the response to TKIs treatment due to the limited number of *EGFR* gene mutant SQCLC patients taking TKIs. Follow-up was done on 13 of the 28 *EGFR* gene mutant cases in our study. Of these, four subjects had died and four subjects were in advanced stages of disease and accepted TKIs treatment. All four acquired clinical benefits from this treatment. Other driving genomic alterations, such as in ALK and c-MET genes, have also been gaining recent attention. However, their mutation rates are lower (ALK: 1.5–6.7% [20-22], and c-MET: 2–4.1% [23,24]). Thus, it is of utmost importance that SQCLC patients have *EGFR* gene mutation examined and accept the TKIs treatment if appropriate.

## Conclusion

*EGFR* mutations are present in pure SQCLC, and are more frequently detected in females and nonsmoker patients. It is important for all patients with SQCLC to have *EGFR* mutation status examined. These patients with activating *EGFR* mutation could accept TKIs treatment.

## Additional files

Additional file 1:
**Approval of the ethic committee.**

Additional file 2:
**The informed consent of patients.**

## Abbreviations

SQCLC: Squamous cell lung carcinoma
EGFR: Epidermal growth factor receptor
H&E: Hematoxylin-eosin
TKIs: Tyrosine kinase inhibitors
NSCLC: Non-small-cell lung carcinoma
ORR: Overall response rate
PFS: Progression-free survival
OS: Overall survival
DCR: Disease control rate
IHC: Immunohistochemical
RR: Response rate
mPFS: Median progression-free survival
