# Supplementary material for: Epidermal growth factor receptor gene mutation status in pure squamous-cell lung cancer in Chinese patients
Source: BMC Cancer. 2015 Mar 1;15:88. doi: 10.1186/s12885-015-1056-9 (PMC4369095; doi:10.1186/s12885-015-1056-9)
Supplement: Additional file 1: — Approval of the ethic committee. [file 12885_2015_1056_MOESM1_ESM.pdf]

上海市胸科医院
上海交通大学附属胸科医院
Shanghai Chest Hospital
Shanghai Chest Hospital Affiliated Shanghai Jiao Tong University

尊敬的张晴医师：

关于您提交的《单纯鳞状细胞肺癌表皮生长因子受体基因（EGFR）突变状态的检测》一文的研究内容与结果，经我院伦理委员会评估，意见如下：

1. 该项目的研究内容与实施过程符合科学研究的伦理学基本原则；

2. 研究中所使用的生物样本为既有标本再利用，未增加受试者的风险与负担，符合免除受试者知情同意的条件。

特此函告。

上海市胸科医院伦理委员会

2013 年 12 月 03 日
