# Supplementary material for: Epidermal growth factor receptor gene mutation status in pure squamous-cell lung cancer in Chinese patients
Source: BMC Cancer. 2015 Mar 1;15:88. doi: 10.1186/s12885-015-1056-9 (PMC4369095; doi:10.1186/s12885-015-1056-9)
Supplement: Additional file 2: — The informed consent of patients. [file 12885_2015_1056_MOESM2_ESM.pdf]

住院病人知情同意书

病员同志：

您好！

为使您更快地了解和适应病区生活，积极配合治疗，我们将有关情况向您告知如下：

一、入院需携带日常生活用品，其他物品如：热水瓶、电饭煲、被子、凳子、椅子等请勿带入病区，尤其不可携带生食物入院。

二、您的床位由医院统一安排，请勿任意调换，以免发生差错。

三、住院期间，请您遵守医院的各项制度，保持病区环境整洁。

四、为配合医务人员诊治，请您与您的家人主动提供真实病史。

五、对无行为能力的病员，家属应服从医院安排，承担监护责任。

六、住院病员请勿私自离院，因擅自离院而发生的一切后果由病家自负。

七、住院病员应遵守医嘱，配合治疗，请勿私自请外院医师诊治或自行用药，如需了解病情或有特殊医疗需求，请与床位医师联系。

八、病员因治疗需用不属上海市医疗保险规定报销范围内的药品，医师应事先告知，由病家签字认可方能使用。自费药品须在我院药房内配，请勿外配。

九、病员因治疗需要输血及使用血制品，医师将事先告知输血或使用血制品可能发生的不良后果，并经病家签字后方能使用；如病家拒绝使用请将书面意见交医师备案。

十、住院期间，病员可以签署授权委托书，委托家属代为行使医疗知情同意权和选择权。

十一、住院期间，在对健康无损害的情况下，病员的生物信息标本可能用于科研，我院将负责保护病员的个人隐私。

十二、每天上午 8:00 –10:00 是病房医生查房诊疗时间，陪护家属及探视病员者一律回避，每天下午 12:00 –14:00 是病人午间休息时间，探视病员一律不准进入病房，为病人创造一个安静舒适的环境。

十三、身高1.2米以下的儿童和患有各种传染病者，不得进入病区。

十四、经医生认为病情需要家属陪侍者，可凭陪客证进入病区，陪护过夜每证限一人，晚20:00之后停止所有病员探视，确保医院住院病人的生命财产安全。

十五、住院前请您交清预付款并保留收据，住院期间遇治疗费用不足请及时补足。

十六、我院实行住院费用“一日清”制度，如您需了解住院期间医疗费用情况，可凭本人社保卡或就诊卡在门诊二楼候诊厅、住院大厅和出入院处进行电脑查询；或在办理出院手续时索取住院费用清单。

十七、病人出院，请于出院当天上午 8:00 后到出院结帐处办理出院手续。

十八、住院期间，请病家配合医院行风建设和反腐倡廉工作，不要向医护人员赠送礼金、礼卡、购物券、物品等“红包”及请客吃饭等。

十九、住院期间，对扰乱医院正常秩序，侵犯医护人员人身安全者，将按卫生部、公安部2001年12号通知处理。

二十、住院期间，如对医院工作有意见或建议，可通过院长信箱或与院长接待办公室联系，投诉电话：62813852

上海市胸科医院

2010 年 4 月

住院病人知情同意书回执

如果您对以上款项表示认可，并自愿入住我院接受诊治，请在下面签名。

签名：沈洪希　　　病区：十　　　2010 年 10 月 17 日
